# Strategy‐Situation Fit of Proactive Emotion Regulation and the Role of Personality

**DOI:** 10.1111/jopy.70040

**Published:** 2025-12-23

**Authors:** Mario Wenzel, Zarah Rowland

**Affiliations:** ^1^ Institute of Psychology University of Wuppertal Germany; ^2^ Institute of Psychology Johannes Gutenberg‐Universität Mainz Germany

**Keywords:** ambulatory assessment, emotion regulation, flexibility, personality, strategy‐situation fit

## Abstract

**Introduction:**

Research on emotion regulation has primarily focused on reactive strategies, while proactive emotion regulation remains less understood. This study investigates proactive emotion regulation through situation selection, where individuals deliberately choose environments to promote desirable emotional outcomes, and examines how this strategy adapts to contextual demands (strategy‐situation fit).

**Methods:**

We reanalyzed an ambulatory assessment dataset comprising 179 participants and 29,956 momentary observations to test preregistered hypotheses about context sensitivity, strategy‐situation fit, and personality predictors.

**Results:**

Results supported our hypotheses: (a) participants showed greater situation selection when experiencing higher‐than‐usual momentary affective well‐being, indicating context sensitivity; (b) this context‐sensitive strategy use was linked to improved overall affective well‐being, demonstrating adaptive strategy‐situation fit; and (c) higher levels of personality plasticity, but not stability, predicted better strategy‐situation fit.

**Conclusion:**

These findings underscore the adaptive potential of situation selection and highlight personality's role, particularly plasticity, in shaping anticipatory regulatory processes. The results inform refinement of emotion regulation theories and suggest avenues for tailoring interventions to individual differences in emotion regulation tendencies.

## 1 Introduction

Emotion regulation research has long focused on finding adaptive ways to modify affective experiences (Gross and Thompson 2007). Extensive studies in both laboratory environments (Webb et al. 2012) and everyday life (Boemo et al. 2022) have shown that although some strategies, such as reappraisal, tend to enhance affective well‐being, other strategies, such as suppression, are generally less effective. However, the overall associations of emotion regulation with affect are often modest and inconsistent (Boemo et al. 2022; Webb et al. 2012). Recent theories emphasize *emotion regulation flexibility* and the alignment of strategies with specific contexts, known as *strategy‐situation fit* (Aldao et al. 2015; Bonanno and Burton 2013). Moreover, individual differences in personality traits may shape one's propensity to select and apply strategies: for example, some people deliberately upregulate negative emotions when it serves a functional goal, such as boosting test performance (Tamir 2005). Despite growing theoretical interest, empirical evidence for these context‐sensitive, trait‐driven processes is still limited with regard to emotion regulation in daily life. To help fill this gap, the present study reanalyzes an ambulatory assessment dataset.

### 1.1 Emotion Regulation Flexibility and Situation‐Strategy Fit in Daily Life

According to Aldao et al. (2015), an emotion regulation strategy is used flexibly when it aligns with contextual variations in an adaptive manner, meaning it is aligned to the demands of the situation. In other words, effective regulation begins with recognizing the environmental demands of a situation (i.e., *context sensitivity*) and selecting the strategy that best fits those demands (Bonanno and Burton 2013), a concept known as strategy‐situation fit (Haines et al. 2016).

Prior research has mainly examined two contextual factors in emotion regulation: situation controllability and affect intensity. For example, Troy et al. (2013) showed that reappraisal is linked to lower depression in uncontrollable situations but may be less effective when control is high, a pattern also seen in daily life (Haines et al. 2016; Wenzel et al., 2020). Laboratory studies further suggest that individuals adapt their emotion regulation strategies based on emotional intensity, often opting for less effortful approaches like distraction when negative affect is high (Sheppes 2020; Troy et al. 2018; Wenzel et al. 2023). Yet, evidence from everyday settings remains mixed, with varying findings on maladaptive (e.g., distraction, rumination, suppression) and adaptive strategies (e.g., reappraisal, acceptance) depending on negative affect intensity (e.g., Blanke et al. 2022; Lennarz et al. 2019; Ortner and Pennekamp 2020; Smith et al. 2023).

These findings indicate that emotion regulation strategy selection may vary with affect intensity. Yet, it remains unclear whether favoring a strategy typically seen as maladaptive, such as suppression, over a more adaptive one like reappraisal, in high‐intensity situations, can still be associated with better well‐being, thus reflecting regulatory flexibility. Evidence linking strategy selection to context sensitivity is limited, and studies have yielded mixed results. One found that using distraction in response to high‐ versus low‐intensity stressors was associated with a significant reduction in negative affect, whereas rumination was less adaptive; the effect of acceptance and reappraisal was unaffected by stressor intensity (Blanke et al. 2022). However, a coordinated data analysis based on 12 ambulatory assessment studies found no evidence for strategy‐situation fit: Choosing distraction in high‐intensity and reappraisal in low‐intensity situations was not significantly associated with positive affect, negative affect, or depressive symptoms (Wenzel and Rowland 2025).

These studies share a common focus on reactive regulation–managing emotions once elicited through flexible in‐the‐moment control (Martins‐Klein et al. 2020). This contrasts with proactive emotion regulation strategies, which involve anticipating and minimizing negative emotions before they arise (Martins‐Klein et al. 2020).

### 1.2 Strategy‐Situation Fit of the Proactive ER Strategy Situation Selection

Building on this distinction between reactive and proactive emotion regulation strategies, situation selection exemplifies a proactive approach by enabling individuals to shape their emotional experiences in advance. This involves evaluating upcoming contexts and intentionally approaching or avoiding certain situations based on their expected emotional impact (Gross 2015). For example, individuals may seek out uplifting events while avoiding stressful situations. By anticipating emotional outcomes and making deliberate choices accordingly, individuals can promote adaptive behavior and overall well‐being.

Specifically, we define situation selection as the intentional act of choosing or avoiding situations based on their anticipated emotional consequences (Webb et al. 2017). This definition is consistent with theories of situation navigation, which conceptualize individuals as active agents who shape their situational experiences through processes such as selection, modification, and construal (Rauthmann and Sherman 2020). From this perspective, situation selection represents one way in which people proactively regulate both their emotional states and their broader person–situation transactions.

There is growing evidence that positive affect may enhance the propensity to engage in proactive emotion regulation. It has been linked to greater cognitive flexibility, creativity, and problem‐solving abilities (Fredrickson 2001), which may facilitate anticipation and context‐sensitive decision‐making. Using the broadened perspective and enhanced resources that come with positive affect, individuals may preemptively reduce the occurrence of challenging emotion regulation situations in the future. Some studies suggest that individuals who experience higher levels of positive affect are more likely to adopt proactive strategies like situation selection, potentially enabling them to generate and sustain beneficial emotional states (Isen 2000). Nonetheless, while these findings are promising, further empirical investigation is needed to fully understand the link between positive affect and proactive emotion regulation.

### 1.3 Individual Differences in Strategy‐Situation Fit of Situation Selection

Lastly, we were interested in individual differences in the strategy‐situation fit of situation selection, particularly as a function of personality traits. Prior research has shown that individuals high in neuroticism may deliberately up‐regulate negative affect (e.g., worry) when doing so serves a motivational purpose, such as preparing for a challenging test (Tamir 2005). These findings suggest that both situational demands and individual motivational tendencies jointly shape emotion regulation processes. Drawing on the Cybernetic Big Five Theory (DeYoung 2015), we focus on the meta‐traits of *plasticity* and *stability*, which represent broad functional dimensions of personality linked to fundamental aspects of goal‐directed behavior and self‐regulation. Plasticity reflects an individual's tendency toward exploration, engagement, and adaptability, encompassing the traits of extraversion and openness. This meta‐trait supports cognitive and behavioral flexibility, motivating individuals to seek out new experiences and actively shape their environments. Stability, in contrast, reflects the capacity to maintain emotional, motivational, and social equilibrium and is characterized by low neuroticism, high conscientiousness, and high agreeableness. It supports consistent self‐regulation by promoting impulse control, emotional resilience, and social harmony.

Because proactive emotion regulation, such as situation selection, involves anticipating and shaping one's emotional landscape in advance of affective events, it may be more closely linked to plasticity than to stability. Individuals high in plasticity are typically more motivated to explore their environment, pursue goals, and adapt proactively to situational demands; their openness and drive for engagement facilitate identifying and selecting situations aligned with their objectives, thereby minimizing the need for reactive regulation. Conversely, individuals high in emotional stability tend to prioritize predictability and the maintenance of familiar routines. This profile facilitates reactive forms of regulation by supporting composure and rapid recovery once emotional challenges arise. However, it provides comparatively fewer advantages for situation selection, which requires anticipating emotional trajectories, evaluating alternatives, and intentionally shaping one's environment before emotions unfold. Prior work indicates that situation selection is most likely to be used, and most effective, among individuals who experience higher emotional reactivity or lower regulatory competence (Webb et al. 2017), suggesting that the motivational impetus for proactive emotion regulation is strongest when emotional volatility is high. Stability, by contrast, is characterized by lower emotional reactivity and reduced perceived need for such pre‐emptive action. Although direct evidence linking emotional stability to lower use of situation selection is limited, reviews of trait–regulation associations highlight that antecedent‐focused, anticipatory strategies rely on cognitive and motivational processes distinct from those supported by stability (Hughes et al. 2020). Together, plasticity and stability may represent complementary resources within the emotion regulation system: plasticity enables proactive exploration and environmental control, whereas stability supports reactive management and emotional equilibrium.

### 1.4 Present Study

Although the importance of strategy–situation fit has been widely discussed in the context of *reactive* emotion regulation, much less is known about its *proactive* counterpart, in the present research exemplified by situation selection. Prior studies on situation selection have shown that individuals can, in principle, shape their emotional experiences by approaching or avoiding situations with anticipated affective consequences (English et al. 2017; Gross 2015). For instance, laboratory work indicates that people often prefer to avoid situations expected to elicit negative emotions and instead select ones likely to induce positive states (Rovenpor et al. 2013). These findings suggest that situation selection is a central but underexplored pathway to adaptive regulation.

However, research to date has mostly examined situation selection at a broad dispositional level or in isolated laboratory tasks, leaving open questions about its role in daily life and its interaction with contextual demands. Closing this gap is critical for two reasons. First, focusing only on reactive processes risks underestimating the regulatory resources individuals mobilize before emotions arise. Second, understanding proactive regulation like situation selection may help explain how people can prevent recurring affective challenges, thereby reducing the burden on reactive strategies, which are particularly effortful to implement (Wenzel et al. 2023). In particular, testing whether proactive regulation demonstrates strategy‐situation fit in daily life is essential for clarifying whether selecting into or out of situations adaptively supports well‐being in ecologically valid contexts.

Moreover, while theories suggest that individual differences such as personality traits should shape how people deploy proactive regulation (Tamir 2005; DeYoung 2015), empirical evidence is still scarce. Existing studies have shown that trait‐like differences predict overall tendencies toward situation selection (e.g., English et al. 2017), but they rarely examine whether these tendencies translate into *context‐sensitive* selection in everyday settings. Thus, linking situation selection to both contextual variation and personality traits moves beyond describing whether and how strongly people select situations to asking *when* and *for whom* situation selection is adaptive.

To address this gap in the present study, we tested three preregistered hypotheses by reanalyzing an ambulatory assessment dataset. We hypothesized that higher levels of momentary affective well‐being would be positively associated with greater use of situation selection, that is, context sensitivity (Hypothesis 1). To demonstrate strategy‐situation fit, we hypothesized that this context‐sensitive use of situation selection would be associated with better overall affective well‐being (Hypothesis 2). Finally, we hypothesized that plasticity, but not stability, would be significantly associated with better strategy‐situation fit (Hypothesis 3).

## 2 Methods

### 2.1 Transparency and Openness

We re‐analyzed an ambulatory assessment dataset to test the hypotheses of the present research. The dataset is available upon request to the EMOTE database, using our data request number (U0X622X9MH). We report all manipulations and exclusions in the present research, but not all measures, because we re‐analyzed the dataset, which was collected to answer a different research question. Thus, sample size planning for the dataset was not based on the objectives of the present research. The hypotheses and the data preparation and analysis scripts to test them were preregistered (https://osf.io/mgpra/) prior to requesting and receiving the dataset and the study materials through the EMOTE database, which can be accessed by the reviewers (https://emote.s3.amazonaws.com/data/downloads/U0X622X9MH_2025‐03‐25FEEL_Study_1.csv?AWSAccessKeyId=AKIA5IJTMFGM2GYI7WUH&Signature=BcWBRrpfiMq0WmrKz3XynTbz%2F%2BA%3D&Expires=1742909099). Thus, the research reported here should be interpreted as confirmatory. The Mplus output can be found on the OSF project page (https://osf.io/qgp7r/). This research was funded by a grant awarded to Mario Wenzel by the German Research Foundation (513283998). In addition, ethical approval for the original study was obtained from the Australian Catholic University Human Research Ethics Committee. Finally, there is no conflict of interest to report.

### 2.2 Participants and Datasets

For the original research purposes, a total of 186 participants were recruited through advertisements distributed via traditional (e.g., printed flyers) and digital (e.g., social media and classifieds websites) media platforms. Eligibility criteria included: (a) being 18 years or older, (b) owning a compatible iOS or Android smartphone for the ambulatory assessment app, (c) fluency in English, (d) access to a computer with a recent version of Windows or macOS, and (e) not currently taking heart or blood pressure medication. Participants were screened for eligibility through a phone call before attending an initial lab session. During this session, one participant was found ineligible and excluded, while six others withdrew during the ambulatory assessment period, resulting in a final sample of 179 adults (65% women; M = 27.0, SD = 9.0). On average, participants responded to 84.0% of the scheduled prompts (SD = 12.7%, Range = 34.9%–100%), completing a total of 29,956 measurement occasions. After the filtering out signals that came after the 21st day and participants who answered less than 33% of the surveys, the datasets included a total of 28,344 measurement occasions. Participants were reimbursed up to AU$150, with the amount partially contingent on their ESM compliance. Ethical approval for the study was granted by the Australian Catholic University Human Research Ethics Committee.

### 2.3 Procedure

The original study consisted of an initial lab session, a 21‐day ambulatory assessment period, and a follow‐up lab session. During the initial session, participants provided informed consent, completed a demographic questionnaire, and filled out several trait self‐report measures. They were instructed to download SEMA2, a smartphone application designed for ESM research (Harrison et al. 2017) and received detailed guidance on the ambulatory assessment procedure.

SEMA2 was programmed to send ambulatory assessment surveys between 10 a.m. and 10 p.m. at intervals of 80 ± 30 min, resulting in approximately nine surveys per day. Each survey expired after 20 min to prevent retrospective completion. While participants were encouraged to complete as many surveys as possible, the researcher emphasized that providing honest and thoughtful responses was more important than maximizing survey completion. Before leaving the lab, participants completed one ambulatory assessment survey with the researcher present to test the ambulatory assessment procedure and were encouraged to ask any clarification questions.

During the 21‐day ESM period, researchers sent weekly emails to update participants on their compliance and maintain engagement. After this period, participants returned to the lab for a follow‐up session, where they completed the trait self‐report measures again.

### 2.4 Measures

Momentary affective well‐being (M = 40.9, SD = 22.9, mean between‐person reliability of $\bar{r_{\mathrm{SB}}}$ = 0.95 based on split‐half reliability (Eisinga et al. 2013)) was assessed by calculating affect balance (Park et al. 2023). Affect balance, calculated as the excess of positive over negative affect, involved subtracting current negative affect from current positive affect. Positive affect was derived from the mean of the items on three positive emotions (confident, happy, and relaxed) and negative affect from the mean of the items on three negative emotions (angry, sad, and stressed), with all items being assessed on a slider that ranged from 0 (*not at all*) to 100 (*very much*).

Situation selection (“I chose which situation to put myself in.”) was assessed at each ambulatory assessment prompt on a scale ranging from 0 (*not at all*) to 100 (*very much*) with respect to “since the last survey”. Situation selection (M = 59.4, SD = 19.9) showed a very good between‐person reliability, $\bar{r_{\mathrm{SB}}}$ = 0.96. The situation selection item reflected the core feature of situation selection as an intentional act of choosing or avoiding situations based on their anticipated emotional consequences. By focusing on the individual's agency in determining which situations to enter or avoid, it captured the proactive and anticipatory nature of situation selection emphasized in conceptualizations of emotion regulation (Webb et al. 2017).

Plasticity and stability were derived from the Big Five Inventory (John et al. 1991), which uses a five‐point scale that ranges from 1 (*disagree strongly*) to 5 (*agree strongly*). Based on Digman (1997), who found a two‐factor structure with one factor related to Agreeableness (M = 3.75, SD = 0.71, ω = 0.57), Conscientiousness (M = 3.49, SD = 0.82, ω = 0.70), and Neuroticism (reversed scored, M = 3.09, SD = 0.57, ω = 0.84) and the second factor related to Extraversion (M = 3.15, SD = 0.63, ω = 0.85) and Openness (M = 3.61, SD = 0.67, ω = 0.48), we took the mean of the respective factors, with higher scores indicating higher plasticity (M = 3.38, SD = 0.49, ω = 0.78) and higher stability (M = 3.44, SD = 0.49, ω = 0.78), respectively.

### 2.5 Analytic Approach

The data was prepared in Stata 17 (College Station, TX, USA: StataCorp LP) and analyzed in Mplus version 8.10. We used a criterion of *p* < 0.05 to assess significance and reported standardized effect sizes. To test the hypotheses, we computed multilevel models using the dynamic structural equation modeling framework in Mplus (Asparouhov et al. 2018).^1^ To ensure that associations among the momentary variables were not artifacts of gradual shifts over the study period, we removed linear time trends from all Level‐1 variables, as preregistered, by regressing each Level‐1 variable on time (i.e., signal number) and using the residuals in all subsequent analyses. This detrending step preserves the within‐person covariation of interest while minimizing bias introduced by slow drifts in mood, context, or compliance across days. This approach is standard in intensive longitudinal analyses where temporal nonstationarity may obscure the processes under investigation (Bolger and Laurenceau 2013).

For preliminary analyses, we wanted to examine the associations of plasticity and stability with situation selection, assuming that plasticity but not stability would be associated with situation selection. To test our hypotheses, we computed a single two‐level model in which, in the within‐level part of the model, prior momentary affective well‐being at measurement occasion *t*‐1 predicted subsequent situation selection at *t*, controlling for prior situation selection at *t*‐1 and allowing for random effects.^2^ The association between prior momentary affective well‐being and subsequent situation selection provided our measure of context sensitivity to test Hypothesis 1, with a positive estimate indicating more context‐sensitive use of situation selection. Specifically, we modeled the lagged association between prior state affective well‐being and subsequent situation selection by allowing the slope of situation selection on lagged affective well‐being to vary randomly across participants. This random slope represents each person's context sensitivity in their use of situation selection (i.e., the extent to which they increase or decrease selection in response to prior affective well‐being), while controlling for the autoregressive effect of prior situation selection.

At the between‐person level, the model examined how individual differences in personality and overall behavior related to context‐sensitive situation selection and overall affective well‐being. First, we modeled overall affective well‐being as a function of both context sensitivity (i.e., the person‐specific random slope linking prior affective well‐being to subsequent situation selection) and individuals' overall tendency to engage in situation selection (their person‐level mean across the study). This specification tested Hypothesis 2, namely, whether people who use situation selection in a more context‐sensitive manner experience higher overall well‐being, above and beyond simply selecting situations more frequently. In addition, context sensitivity was regressed on plasticity and stability in the same model, allowing us to test Hypothesis 3: whether individuals higher in these metatraits tend to use situation selection in a more or less context‐sensitive manner.

We identified one deviation from the preregistered analyses: two Big Five Inventory items had been mistakenly preregistered as reverse‐scored. In the revised analyses, we use the correctly scored items, and the exact item wording and intended scaling are provided on the OSF project page (https://osf.io/qgp7r/).

## 3 Results

Before testing our hypotheses, we examined the general, that is, context‐insensitive, associations between the study variables. First, situation selection was not significantly associated with either plasticity, β = 0.14, 95% CI [−0.01, 0.28], or stability, β = 0.07, 95% CI [−0.07, 0.22]. Second, overall affective well‐being was significantly associated with both plasticity, β = 0.20, 95% CI [0.06, 0.32], and stability, β = 0.32, 95% CI [0.19, 0.44], as well as with situation selection, β = 0.29, 95% CI [0.14, 0.41], with the latter demonstrating the general benefit of selecting situations.

### 3.1 Registered Analyses

Hypothesis 1 **Context sensitivity**. *We found a significant association between prior momentary affective well‐being and subsequent situation selection,* β *= 0.06, 95% CI [0.04, 0.07], indicating that the better than usual participants felt, the more they used situation selection. Thus, we found support for Hypothesis* *1* *regarding the context‐sensitive use of situation selection*.

Hypothesis 2 **Strategy‐situation fit**. *Hypothesis* *2* *tested whether the context‐sensitive use of situation selection was actually adaptive for the overall affective well‐being, which was the case,* β *= 0.23, 95% CI [0.01, 0.44]. Participants who used situation selection more than usual in situations in which they felt better than usual reported better overall affective well‐being than participants who used situation selection less contextually. Thus, we also found support for Hypothesis* *2* *regarding the strategy‐situation fit of situation selection*.

Hypothesis 3 **Strategy‐situation fit and plasticity and stability**. *In Hypothesis* 3, *we examined individual differences in strategy‐situation fit of situation selection. The results showed that plasticity was significantly associated with better strategy‐situation fit,* β *= 0.27, 95% CI [0.04, 0.45], while stability was not,* β *= 0.11, 95% CI [−0.11, 0.31], providing support for Hypothesis* 3.

### 3.2 Updated Analyses

In addition, non‐preregistered analyses, we refined our operationalization of both the metatraits and context‐sensitive situation selection. First, instead of relying on affect balance, we modeled context sensitivity separately for positive and negative affect. Because strategy–situation fit may be influenced by individuals' average affective levels and their overall tendency to engage in situation selection, we residualized the positive‐ and negative‐affect context‐sensitivity indices by regressing each on person‐level mean situation selection and person‐level mean positive or mean negative affect, respectively. The resulting residualized indicators therefore reflect the extent to which individuals adjust their use of situation selection as a function of momentary affect, independent of general selection tendencies or typical affect levels.

The two residualized indicators were strongly correlated (*r* = 0.64) when negative‐affect context sensitivity was reverse‐scored, suggesting that they captured a shared underlying process. Accordingly, we defined a latent *context‐sensitivity* factor indicated by residualized positive‐affect context sensitivity and reverse‐scored residualized negative‐affect context sensitivity, with factor loadings constrained to equality to stabilize estimation and prevent one indicator from disproportionately influencing the latent construct. This factor demonstrated acceptable reliability (McDonald's ω = 0.80), indicating systematic individual differences in affectively informed situation selection.

Next, to more precisely capture the metatraits of plasticity and stability, we also adopted a latent‐variable approach for them. Plasticity was modeled as a latent factor indicated by extraversion and openness (with loadings constrained to equality), and stability was modeled as a latent factor indicated by conscientiousness, agreeableness, and reverse‐scored neuroticism. This approach isolates the variance common to the traits within each metatrait and avoids inflating metatrait scores with trait‐specific variance.

Using this latent framework, plasticity was significantly associated with more context‐sensitive situation selection, β = 0.43, 95% CI [0.08, 0.78],^3^ consistent with and stronger than the preregistered association observed using the aggregate metatrait scores. In contrast, stability was again not significantly related to context sensitivity, β = 0.06, 95% CI [−0.16, 0.27]. These findings indicate that individuals higher in plasticity, but not stability, tend to select situations in a manner that more adaptively reflects their momentary affective context.

To further clarify the personality dimensions underlying these associations, we examined each of the Big Five traits as predictors of the latent context‐sensitivity factor. Openness to experience showed a significant positive association with context‐sensitive situation selection, β = 0.19, 95% CI [0.04, 0.35]. Extraversion showed a smaller, non‐significant positive association (β = 0.09, 95% CI [−0.07, 0.25]), which was larger than the remaining traits, which loaded onto stability, conscientiousness (β = 0.01, 95% CI [−0.15, 0.17]), agreeableness (β = 0.07, 95% CI [−0.09, 0.23]), and neuroticism (β = −0.04, 95% CI [−0.21, 0.13]). Although only openness reached statistical significance, the two traits associated with the plasticity metatrait (openness and extraversion) exhibited the largest effect sizes in the expected direction, supporting the interpretation that plasticity‐related characteristics play a central role in explaining individual differences in context‐sensitive situation selection.

## 4 Discussion

The present study examined the strategy‐situation fit of proactive emotion regulation by investigating whether situation selection is used in a context‐sensitive manner and whether such use is associated with better affective well‐being. Additionally, we explored whether individual differences in plasticity and stability predict better strategy‐situation fit. Our findings provide novel insights into the dynamics of proactive emotion regulation in daily life and highlight important theoretical and empirical implications for understanding adaptive emotion regulation.

### 4.1 Context Sensitivity and Strategy‐Situation Fit of Situation Selection

We found support for Hypothesis 1, demonstrating that individuals used situation selection in a context‐sensitive manner: Participants were more likely to select their situations when experiencing higher momentary affective well‐being. This suggests that individuals may leverage positive affect to proactively shape their environment, consistent with the broaden‐and‐build theory of positive emotions (Fredrickson 2001). Positive affect has been associated with increased cognitive flexibility and goal‐directed behavior, which may facilitate anticipatory emotion regulation processes such as situation selection (Isen 2000). Our findings extend this literature to the emotion regulation literature by showing that such tendencies are evident in real‐world regulatory contexts.

Moreover, Hypothesis 2 was also supported: Individuals who used situation selection in a context‐sensitive manner reported greater overall affective well‐being. These findings suggest that adapting situation selection with momentary affective states may have cumulative benefits for well‐being. This is consistent with previous research suggesting that adaptive emotion regulation involves aligning strategies with situational demands (e.g., Bonanno and Burton 2013; Haines et al. 2016). While previous studies have primarily focused on reactive emotion regulation strategies (e.g., reappraisal, suppression), our findings suggest that situation selection may follow a similar principle. Specifically, situation selection appears to be most beneficial when individuals are in a positive mood, highlighting the importance of strategy‐situation fit in both proactive and reactive emotion regulation.

At the same time, alternative interpretations should be acknowledged. For instance, individuals experiencing higher positive affect may *perceive* themselves as having greater control over their environment, even if their objective control is unchanged. Conversely, individuals who generally experience greater control in daily life may do so for reasons unrelated to emotion regulation and consequently report higher positive affect. Future research using more objective measures of control or momentary assessments of perceived agency could help disentangle these possibilities.

Additionally, an important open question concerns the mechanism underlying these benefits: Do individuals feel better because they exercised agentic choice, thereby enhancing a sense of control, or because the chosen situations themselves^4^ are more emotionally rewarding? For example, someone may feel better after choosing to go to a café either because the act of choosing reinforces autonomy or because the café visit itself provides positive stimulation. Future studies that differentiate these pathways (e.g., comparing self‐chosen vs. assigned situations) could clarify whether the affective benefit arises primarily from agency, situational quality, or their interaction.

### 4.2 Individual Differences in Strategy‐Situation Fit

Consistent with Hypothesis 3, plasticity predicted better strategy–situation fit in situation selection, whereas stability did not. This supports the view that situation selection is more closely tied to exploration, engagement, and openness to new opportunities—core characteristics of plasticity (DeYoung 2015). Plasticity reflects a tendency toward active engagement with the environment, curiosity, and readiness to explore potentially rewarding contexts. These qualities naturally facilitate proactive attempts to shape one's emotional landscape before challenges arise, making plasticity well suited to the demands of situation selection. Importantly, the present findings highlight how higher‐order personality dimensions channel individuals toward distinct modes of self‐regulation. Plasticity has often been described as a motivational and cognitive orientation toward possibility: seeking information, testing boundaries, and approaching situations with a readiness to revise one's course (DeYoung 2015). Our results suggest that this orientation does not merely characterize how people respond to the world; it also shapes how they anticipate it. Individuals higher in plasticity seem more likely to use their current affective states as signals for adaptive action and to select into situations that may sustain or enhance their well‐being. In other words, proactive regulation may represent one behavioral manifestation of the exploratory, agentic tendencies that define plasticity.

These findings also deepen our understanding of the role of personality in emotion regulation flexibility. Much work on flexibility treats regulatory behavior as driven primarily by situational cues and learning processes (Aldao et al. 2015; Bonanno and Burton 2013; Kashdan and Rottenberg 2010), but the present results indicate that personality traits help determine who is most capable of deploying flexible strategies. From this perspective, plasticity functions as a resource that supports context‐sensitive regulation: the motivational drive associated with extraversion, together with the curious and open stance characteristic of openness to experience, may amplify the broadening effects of positive affect. This combination may help individuals scan their environment for opportunities, generate alternative behavioral paths, and act on those possibilities before problems arise. Personality psychology therefore contributes a crucial layer to theories of emotion regulation by showing that flexibility is not only domain‐dependent or a skill but that it is also partly dispositional.

In contrast, stability did not significantly predict strategy–situation fit. Stability reflects emotional evenness, impulse control, and the capacity to maintain goal‐directed functioning without being easily disrupted (DeYoung 2015). These characteristics may contribute more strongly to reactive emotion regulation, for example, helping individuals remain composed once challenges occur, than to proactive strategies that require anticipatory engagement with one's environment. From a cybernetic perspective, stability supports the maintenance of existing goals and behavioral patterns, whereas plasticity facilitates updating and exploration. The null finding for stability therefore underscores the differentiation between maintaining versus reshaping the person–situation interface.

These results also highlight an important conceptual nuance for personality psychology: not all adaptive outcomes associated with emotion regulation are grounded in the same personality foundations. Stability has been linked to lower negative affect, greater emotional resilience, and more effective recovery from stress (e.g., Ormel et al. 2012), but our findings suggest that these advantages do not necessarily extend to anticipatory, opportunity‐seeking forms of regulation. Instead, proactive strategies may be a hallmark of individuals who are dispositionally inclined to intervene earlier in emotional trajectories, before distress arises. This distinction enriches existing models by suggesting that the personality system may support emotional adaptation through multiple pathways, one rooted in maintaining equilibrium (stability) and another rooted in actively shaping the environment (plasticity).

Finally, by examining meta‐traits rather than individual traits alone, the present study underscores the value of higher‐order personality structure for understanding emotion regulation in daily life. The absence of associations with stability suggests that the mechanisms supporting proactive regulation may be more closely tied to exploratory cognition and motivational engagement than to self‐control or emotional steadiness per se. This finding encourages future work to investigate whether proactive and reactive regulation form parallel pathways that correspond to the personality architecture reflected in plasticity and stability. Such a framework would enrich theories of personality dynamics by linking the structural organization of traits to real‐time regulatory behaviors in everyday life.

### 4.3 Practical Implications

Taken together, the present findings have important implications for theories of emotion regulation, particularly regarding the role of the proactive strategy situation selection in promoting well‐being. Most research on emotion regulation has focused on reactive strategies, but our study highlights the need to further investigate proactive regulation. Given that situation selection was most effective when used in a context‐sensitive manner, interventions aimed at improving emotion regulation might benefit from incorporating practice in situation selection. For example, individuals could be taught to identify moments when they are best equipped to make proactive choices and use their high momentary affective well‐being to optimize future emotional outcomes. In addition, our findings highlight the importance of considering personality traits in emotion regulation interventions. Individuals high in plasticity may be more inclined to engage in situation selection, while those high in stability may benefit from other strategies. Tailoring interventions to individuals' regulatory tendencies may increase their effectiveness and improve long‐term well‐being. In addition, understanding whether the emotion‐regulatory value of situation selection derives primarily from feelings of agency or from exposure to rewarding contexts may further guide intervention efforts by clarifying whether techniques should focus on enhancing perceived control, expanding access to positive environments, or both.

### 4.4 Limitations and Future Directions

While our study provides valuable insights, several limitations should be acknowledged. First, our analyses relied on ambulatory assessment data, which provide high ecological validity but do not allow for causal conclusions. Experimental studies based on the emotion regulation choice (e.g., Sheppes et al. 2011) that manipulate affective states and examine subsequent regulatory choices could provide evidence for the causal role of positive affect in situation selection and in proactive emotion regulation in general. Second, although our study captured real‐world emotion regulation dynamics, it focused on a single proactive strategy, situation selection. Future research should examine whether other proactive strategies, such as social approach behaviors, mastery pursuits, or efforts to structure daily contexts, follow similar patterns of strategy‐situation fit. Third, our sample was predominantly young adults, which may limit the generalizability of our findings. Given that age‐related differences in emotion regulation strategies have been documented (Urry and Gross 2010), future studies should explore whether older adults exhibit similar patterns of proactive emotion regulation flexibility. Fourth, we acknowledge that the item for situation selection (“I chose which situation to put myself in”) does not explicitly reference the emotional consequences of the chosen situation. From a conceptual standpoint, this could be seen as a limitation, as definitions of situation selection typically emphasize the anticipatory regulation of emotions through situational choices (Webb et al. 2017). In addition, the item was assessed retrospectively, after individuals had already chosen a situation, meaning that the self‐reported regulation may have been biased and not accurately reflect its proactive nature. However, we view this item as a reasonable and face‐valid indicator of the behavioral core of situation selection; namely, the intentional act of choosing or avoiding situations. While it does not explicitly capture the emotional motivation underlying these choices, it reflects the agentic component central to this strategy. Future research could benefit from refining this assessment by incorporating explicit reference to the anticipated emotional impact of situational choices to more directly align with theoretical definitions of situation selection as an emotion regulation process.

Finally, a further limitation of the present study is the reliance on self‐report measures for both personality and daily contextual processes. Self‐reported momentary states often share variance due to general reporting styles or mood‐congruent responding, which raises the possibility that some associations may be influenced by method factors rather than substantive psychological processes (Podsakoff et al. 2003). At the same time, several features of our findings suggest that broad response tendencies are unlikely to fully account for the results. In particular, the pattern of associations was highly selective: traits linked to plasticity showed meaningful relations with context‐sensitive situation selection, whereas traits associated with stability showed little or no association. This heterogeneity is difficult to explain by a general response bias, which would be expected to produce more uniform associations across traits and outcomes. Nonetheless, the use of a single assessment method remains an important constraint, and future research would benefit from incorporating behavioral indicators, informant reports, or other multimethod approaches to better disentangle substantive associations from potential method variance.

## 5 Conclusion

The present study extends research on emotion regulation flexibility by demonstrating that situation selection follows principles of strategy–situation fit: Individuals were more likely to engage in this proactive strategy when experiencing positive affect, and such context‐sensitive use was associated with greater well‐being. Individual differences in plasticity, but not stability, further shaped these processes, underscoring the importance of considering both situational and personality factors in proactive emotion regulation. By clarifying alternative mechanisms, considering perceived versus actual control, and distinguishing the roles of plasticity and stability, the findings highlight the complexity of proactive regulation and point to promising directions for future research.

## Author Contributions

Mario Wenzel conceptualized the study, conducted the analyses, and led the interpretation of the results. Mario Wenzel drafted the initial manuscript and Zarah Rowland contributed to writing and editing the manuscript. Both authors approved the final version of the manuscript.

## Funding

This research was funded by a grant awarded to Mario Wenzel by the German Research Foundation (513283998).

## Conflicts of Interest

The authors declare no conflicts of interest.

## Data Availability

The data that support the findings of this study are available in EMOTE database at https://emote.s3.amazonaws.com/data/downloads/U0X622X9MH_2025 03 25FEEL_Study_1.csv?AWSAccessKeyId=AKIA5IJTMFGM2GYI7WUH Signature=BcWBRrpfiMq0WmrKz3XynTbz%2F%2BA%3D Expires=1742909099, reference number U0X622X9MH. These data were derived from the following resources available in the public domain: EMOTE database, https://emotedatabase.com/

## Endnotes

^1^
All models used a Bayesian estimator with two MCMC chains, the default Gibbs sampler, default priors so that the results were driven by the data, a thinning of 20, and a convergence cutoff of a potential scale reduction = 1.005. A thinning value of 20 means that out of 20 iterations of the MCMC chains, only 1 is saved. Thus, instead of running at least 2000 iterations per chain, Mplus ran 20 × 2000 = 40,000 and saved every 20th iteration, which reduces issues with possible autocorrelations. Moreover, we required a minimum of 2000 saved iterations before model estimation was terminated in case the convergence cutoff was met to ensure and check for a stable potential scale reduction. After model termination, we visually inspected the models for signs of misspecification, i.e., unstable potential scale reduction values, large (*r* > 0.20) autocorrelations, and trace plots that did not resemble a large, hairy caterpillar. However, we did not find any signs of misspecification and thus included all estimates in the meta‐analytic synthesis.

^2^
Note that Mplus automatically uses latent variable centering in the within‐part (Asparouhov and Muthén 2019), and thus estimates at this level are centered on the latent person mean.

^3^
Results did not differ when using residualized positive‐affect context sensitivity, β = 0.41, 95% CI [0.07, 0.74], or residualized, reverse‐scored negative‐affect context sensitivity, β = 0.38, 95% CI [0.05, 0.71].

^4^
In addition, because context sensitivity was also operationalized using residualized indicators that remove variance associated with mean affect and overall situation‐selection frequency, these findings indicate that the association between plasticity (and particularly openness) and adaptive strategy–situation fit cannot be attributed to individuals simply selecting more situations overall or experiencing more favorable affect on average. Rather, the results point to meaningful personality‐linked differences in how individuals use situation selection in response to affective cues.
